# How cognitive neuroscience could be more biological—and what it might learn from clinical neuropsychology

**DOI:** 10.3389/fnhum.2014.00541

**Published:** 2014-07-21

**Authors:** Stefan Frisch

**Affiliations:** Department of Neurology, University Hospital Frankfurt/Goethe-UniversityFrankfurt am Main, Germany

**Keywords:** clinical neuropsychology, kurt goldstein, localization, embodiment, psychological experiments, critical neuroscience

## Abstract

Three widespread assumptions of Cognitive-affective Neuroscience are discussed: first, mental functions are assumed to be localized in circumscribed brain areas which can be exactly determined, at least in principle (*localizationism*). Second, this assumption is associated with the more general claim that these functions (and dysfunctions, such as in neurological or mental diseases) are somehow *generated inside* the brain (*internalism*). Third, these functions are seen to be “biological” in the sense that they can be decomposed and finally explained on the basis of elementary biological causes (i.e., genetic, molecular, neurophysiological etc.), causes that can be identified by experimental methods as the gold standard (*isolationism*). Clinical neuropsychology is widely assumed to support these tenets. However, by making reference to the ideas of Kurt Goldstein (1878–1965), one of its most important founders, I argue that none of these assumptions is sufficiently supported. From the perspective of a clinical-neuropsychological practitioner, assessing and treating brain damage sequelae reveals a quite different picture of the brain as well as of us “brain carriers”, making the organism (or person) in its specific environment the crucial reference point. This conclusion can be further elaborated: all experimental and clinical research on humans presupposes the notion of a situated, reflecting, and interacting subject, which precedes all kinds of scientific decomposition, however useful. These implications support the core assumptions of the embodiment approach to brain and mind, and, as I argue, Goldstein and his clinical-neuropsychological observations are part of its very origin, for both theoretical and historical reasons.

*In pathologischen Fällen haben wir es etwa beim Aphasischen nicht mit einem Menschen mit veränderter Sprache zu tun, sondern mit einem veränderten Menschen, dessen Veränderung sich uns in Veränderungen seiner Sprache, aber auch in den verschiedensten anderen Erscheinungen kundtut. Also betrachte man nie eine Erscheinung isoliert vom ganzen kranken Menschen*.

[In pathological cases, such as aphasia, we do not deal with a human individual with changes in language but we deal with changes in a human individual, which manifest as changes in language, but also in a range of further phenomena. Thus, never investigate a phenomenon in isolation from the context of the diseased individual (my transl.)]

(Goldstein, 1927, p. 630)

## Outline: three weighty assumptions too rarely questioned

There are at least three widespread assumptions in Cognitive-affective Neuroscience (CNS) that seem to guide many research efforts, knowingly or unknowingly. The first is *localizationism*: functions are assumed to be localized in circumscribed brain areas which can be clearly identified. In this way, even complex functions can be decomposed and related to specific parts of the brain (Kandel, 1998; Price and Friston, 2002; Ardila and Bernal, 2007; Kandel et al., 2013) and the brain essentially exhibits a modular organization (Friston and Price, 2011). Thus, by determining the role of each brain area experimentally, we finally gain a complete understanding of how the brain works, at least in principle. The second idea is entailed in the first, but not vice versa, so it will be treated as a separate claim: *internalism*. Here, it is assumed that cognitive functions are somehow causally *produced inside* brains (Crick and Koch, 2003; Kandel et al., 2013). Understanding and reconstructing the computational principles of the brain (even *in silico*), can reveal how mental functions are actually generated (Kandel et al., 2013) and how mental disorders can be conceived of as brain products as well (Kandel, 1998; Insel and Quirion, 2005; Kandel et al., 2013). The third assumption I will call *isolationism* in order to highlight a fundamental methodological strategy: all scientific knowledge about mind and brain must be derived from controlled experiments in which the investigated phenomenon is isolated under laboratory conditions. The resulting effects can then be put together into a broader picture. In this way, the experimental method is “… a straightforward, conservative extension of objective science that handsomely covers … *all* the ground” of mental phenomena (Dennett, 2003, p. 19).

In this context, clinical neuropsychology is mainly seen as a source of evidence in favor of the assumptions just described, especially by contributing evidence for lesion-deficit correlations. According to this view, clinical neuropsychologists objectively identify deficits by means of standardized tests which they then relate to lesion sites in the brain, in order to provide evidence for the *causal* role of a specific brain area. Most importantly, this complements experimental imaging studies which can only provide *correlative* evidence for brain-function-relationships (Pascual-Leone et al., 1999; Price and Friston, 2002; Ardila and Bernal, 2007; Friston and Price, 2011). Deficits are mainly seen as quantitative changes in performance relative to the mean of a group of controls (Price and Friston, 2002; Noppeney et al., 2004). Such “clinical data” can then be used to reconstruct or even simulate how deficits are produced by diseased or lesioned brains (Kandel et al., 2013), in order to then enable the clinician to manipulate the respective brain mechanisms accordingly.

Certainly, there are cases in which working with lesion data based on these assumptions is adequate. However, generalizing them creates a biased, oversimplified and finally distorted picture of what clinical neuropsychologists actually do and of how brain lesions (and by extension: brains) work. When looking from the perspective of a clinical practitioner, a very different picture of the brain emerges, as I will argue, a picture which is much less compatible with the three above assumptions as may be expected. The German neurologist Kurt Goldstein (1878–1965) shall serve as my principal protagonist, for two reasons: first, Goldstein co-developed the first thorough psychological studies on brain damaged patients (Gelb and Goldstein, 1920). He can therefore count as a founder of modern clinical neuropsychology (Luria, 1966) and of an approach to the patient which I find most appropriate for reasons which I will elaborate on below. Second, as a disciple of Carl Wernicke, Goldstein grew up in the mechanist and localizationist thinking of 19th century aphasiology, but profoundly changed his views on the brain in the course of his diagnostic and therapeutic work with countless brain-damaged WW1 soldiers. Retracing Goldstein’s motives for his radical turn reveals interesting insights that may have considerable relevance for today’s neuroscientific research, despite their age.

## Goldstein’s turn: a more appropriate view of brain lesions (and brains)

The idea that mental abilities are specifically located in circumscribed brain areas dates back to antiquity, but gains considerable prestige in the “classical” period of neurology in the 19th century, where it becomes the dominating—though never undisputed—view on the structure-function relationship. Goldstein’s supervisor Carl Wernicke (1848–1905) assumed that complex functions such as language could be decomposed into elementary mechanisms that were specifically localized in the brain. The classical method was to describe the clinical signs and to relate them to lesion sites which were determined post mortem in most cases. These different observations were then assembled into models of function. In the case of language, “motor images” of words were assumed to be stored in the left inferior frontal gyrus (Broca’s area) and “sound images” of words in the left superior temporal gyrus (Wernicke’s area). Connections between the two areas were seen as connecting speech sounds to speech movements, e.g., when repeating words. Thus, lesions could not only affect the different types of representation, but also the connections between them, thereby producing the different types of aphasic signs (production, comprehension, repetition etc.). Even though this research method became quite successful, it had prominent opponents, such as John Hughlings Jackson (1835–1911) and Constantin von Monakow (1853–1930).

As Wernicke’s disciple, Goldstein initially stood under the influence of a localizationist view. However, as the director of the *Institut für die Erforschung der Folgeerscheinungen von Hirnverletzungen* (*Institute for Research on the Aftereffects of Brain Injury)* at Frankfurt University from 1915 on, where he was responsible for the assessment and treatment of brain-damaged WW1 veterans, he ultimately changed his views. Goldstein (1926, 1934) criticized the localizationist’s notion of “clinical sign” which he found was misconstrued as a transparent, steady, and monolithic phenomenon. By contrast, Goldstein argued that such an approach of just *quantifying* isolated phenomena was artificial in a way as it did not account for *qualitative* aspects of the—organismic/personal and situational—context, for several reasons: first, the patient does not display a loss of function in the transparent way a machine does (e.g., pressing a switch does not illuminate a bulb anymore), but tries to give the best possible *answers* to the *questions* posed by the examiner. Thus, responses are bound to the interactional and situational context of the examination. Second, clinical signs are not only dynamic (i.e., changing) in a diachronic perspective (von Monakow), but they may also change depending on the situational context (Hughlings Jackson). As an example, patients may be able to produce one and the same word in a concrete, real-world situation (e.g., conversation, praying, swearing etc.) but not in the clinical examination where they have to abstract from the usual usage (e.g., when naming pictures). These problems may point to more basic, global disturbances (e.g., in abstraction) which may surface in a range of different phenomena. In relation to this, one and the same clinical sign may come about in very different ways across different situations and/or patients. Thus, a thorough (psychological) analysis of *how* a response was produced would be at least as important as the finding *that* it was produced (see also Goldstein and Scheerer, 1941).

Goldstein showed that the localizationists’ claims were repeatedly “confirmed” not only because their investigations were guided by implicit, *a priori* assumptions (i.e., that clinical signs are context-independent and localized in circumscribed areas in the brain) but also by explaining away contradictory evidence. According to his own observations, focal brain lesions almost never led to a unidimensional outcome with one type of performance (e.g., language production) being completely lost, while all other capacities remained completely unaffected. Instead of interpreting this just as a result of methodological insufficiencies in specifying the deficit and/or the lesion, however, Goldstein called the localizationists’ machine-like model of the brain into question. He concluded that it was inappropriate for the explanation of biological phenomena. He demonstrated that a more comprehensive description of brain damage sequelae revealed changes on different levels: brain injured patients not only lose specific abilities while keeping others, but the whole range of abilities is modified. Responses are becoming “amorphous”, i.e., less differentiated, and organism and environment enter a new relationship (new *quality* or *Gestalt*). Accordingly, the outcome of a brain damage can only be adequately captured in the context of the whole organism, which tries to reach a new arrangement with its environment in order to preserve and maximize its possibilities. This can even be observed in the case of seemingly “basic” functions such as vision, as Goldstein argued by making reference to the work of one of his assistants on the so-called *pseudofovea* (Fuchs, 1922). Fuchs had reported the case of a veteran whose calcarine fissure (primary visual cortex) in one hemisphere had been completely destroyed and who had developed a contralateral homonymous hemianopia (loss of one half of the visual field) as demonstrated in a visual field test (perimetry). In a series of experiments, Fuchs demonstrated that a new site of sharpest vision had developed that differed from the anatomical macula and varied depending on properties of the outside scenery (distance and size of objects, viewing angle etc.). When the patient had to indicate the sharpness of adjacent symbols, the point of maximum focus was not reported as being located at the edge of the visual field, as it should have been according to the perimetry. Instead, it dynamically shifted towards the center of the remaining visual field where it was surrounded by a periphery of decreasing sharpness, as it was familiar to the patient. This *pseudofovea* had developed unconsciously and relatively fast, and thus could not be explained by explicit training. Goldstein interpreted this as a result of the organism’s tendency to try to accomplish tasks in its environment in the usual way as long as possible (Goldstein, 1934).

Here, “environment” is not synonymous with external world in the sense of an ecological niche. Goldstein was influenced by ideas of the biologist Jakob von Uexküll (1864–1944) who promoted the term *Umwelt* (literally: “the world around”, often translated as “subjective world/universe”) in order to describe those parts of the physical world that a specific organism has access to and that are *meaningful* to it. As an example, my terrace can be taken as some kind of independently existing, physical object. Nevertheless, it constitutes fundamentally different *Umwelten* for different organisms, such as me, a bird and a fly, as it provides disparate networks of meaningful (and mostly incommensurable) relationships for the three species (Magnus, 2008; Stjernfelt, 2011). Each organism succeeds “in shaping its environment” (Goldstein, 1934, p. 85) through a permanent interaction with its *Umwelt*, which also includes social relations (ibid., p. 338). The concept of *Umwelt* becomes crucial for Goldstein’s conception of what happens in the case of brain damage: damage to the brain leads, above all, to discrepancies between the organism’s abilities and the demands of its *Umwelt* due to the abrupt and drastic changes that cannot be adjusted for. This unsettles the organism in an existential way, as it had been used to a close and never challenged union with its *Umwelt*. The organism is longing for balance. It can achieve this, mostly unconsciously, in multiple ways: by changes in self-awareness (especially, awareness of the deficit), by reducing its *Umwelt* in order to avoid external demands that cannot be met, by seeking increased control over the environment (through orderliness, rigidity etc.), by compensating by means of other abilities, to name only a few.

Thus, the damage does not just take something from the organism, but the organism reacts to the changes and a new relationship to the *Umwelt* develops, leading to modifications on both sides. These multiple, interrelated changes reveal that organism and *Umwelt* are inextricably intertwined as well as constantly re-established through activity. This idea seems to anticipate the concept of *autopoiesis* (Magnus, 2008, p. 390), especially in its later versions (such as in Varela, 1997; see also Thompson, 2007).

Brain damage profoundly disturbs the organism-*Umwelt* coupling and the organism’s general tendency to preserve an identity. Therefore, it provides a very interesting instance in which these processes come to the fore and can be studied. On the other hand, brain damage itself can be understood in its full range only by adopting a perspective *beyond* the brain itself, namely in a brain-organism-*Umwelt* context. In contrast to many competing attempts to fully decompose properties of life into basic, explanatory units, such as reflexes, words, neurons etc., Goldstein (1934) advocated a systems-based view. He proposed that the concept of *organism* was indispensable to understand processes on the level of the organism’s parts. Such views had and have many prominent successors down to the present day (e.g., Varela, 1997; Thompson, 2007), but Goldstein (1934) can take the credit for providing decisive evidence from clinical neuro(psycho-)logical observations.

## The winner is … localizationism?

Today, the idea of functional localization seems to be largely confirmed by clinical as well as experimental results from modern neuroimaging techniques (Price and Friston, 2002; Ardila and Bernal, 2007; Friston and Price, 2011; Kandel et al., 2013). Thus, Goldstein’s arguments against localizationism seem hopelessly outdated at first glance. However, they still carry significance today. Certainly, I do not want to suggest that 21st century CNS is at the same level as 19th century neurology, neither empirically nor conceptually. Nevertheless, at least two of Goldstein’s points seem worth mentioning: first, “localizing” *clinical signs* is not the same as localizing *functions*, and similarly, “localizing” *experimental effects* is not the same as localizing *functions* either. An experimental effect is—like a clinical sign—a complex, context-dependent entity which entails a range of non-factual decisions on the side of the investigator (choice of paradigm, statistics, true effect vs. error etc.) as well as variables on the side of the subject (strategies, test anxiety, reward etc.). Although some of these can be investigated and controlled for systematically, not all of them can, at least not simultaneously. Second, and more importantly, the idea of modularity as a principle of organization of mind and brain (Friston and Price, 2011) seems so pervasive that it appears in many guises, even at the risk of immunization (van Orden et al., 2001), and despite interesting alternatives.

As soon as neuroimaging methods were mastered sufficiently, research started to map mental functions onto the brain (Kosik, 2003). CNS, especially in its earlier stages, was largely dominated by mapping functions in a quasi-phrenological manner (Bates and Dick, 2000; McIntosh, 2000). The idea of the brain as a collection of specialized areas was hardly disputed, since a variety of studies with focal activations in specific tasks seemed to confirm it. It was even taken for granted that function-region associations could be inferred in both ways, also from activation to function (*reverse inference*, cf. Poldrack, 2006, 2011). Inconsistencies between studies on the same issue soon arose, be it that one and the same experimental paradigm was found to lead to different patterns of activation or that one and the same area was activated in a variety of disparate tasks (Poldrack, 2006, 2011; Anderson and Finlay, 2014). However, these inconsistencies were often interpreted as methodological insufficiencies which could, sooner or later, be overcome by more sophisticated experimental designs or finer anatomical subdivisions (Kosik, 2003). The underlying localizationist assumptions were thereby perpetuated. Even when talking of “networks”, the latter seemed to be conceived of as ensembles of brain areas, each housing an elementary cognitive function, such as phoneme discrimination being important for different tasks such as reading, writing, spelling etc. Finally, the idea behind these networks was “an extension of […] modular theories of cognition” (Ardila and Bernal, 2007, p. 937). Although the types of function to be localized were much more specific and theoretically elaborated, it seemed still like “the 19th century search for isomorphic mappings” (Bates and Dick, 2000, p. 21), with the old mental faculties being dissipated and re-conceptualized on a lower level (McIntosh, 2000; van Orden et al., 2001). In this respect, clinical neuropsychology was welcome to provide *causal* evidence for this idea in the form of lesion-deficit-correlations (Price and Friston, 2002; Ardila and Bernal, 2007). Damage, according to this view, leads to a selected failure of the specific tools that had been provided by the area(s) now lesioned (Kanwisher, 2006).

However, if we follow Goldstein (1934) above advice and do not just focus on isolated, theoretically interesting hypotheses, but dare a broader perspective, we see that there is hardly any lesion that affects one function completely while sparing everything else. The sequelae induced even by seemingly focal changes can indeed be manifold and dynamic (Prinz, 2006), including global changes in overall behavior or personality. In addition, the brain may compensate for a number of initial difficulties, at least in the long run. This can even be seen in sudden lesions, such as strokes, but may be very striking in lesions on a longer time-scale, such as degenerative diseases (Riley et al., 2002) or slow growing tumors (Desmurget et al., 2007). Such tumors very often become first apparent through seizures rather than cognitive deficits, even if they have the size of several centimeters (ibid.). Tumor growth often seems to lead to internal shifts even of primary (e.g., motor) functions which may then be widely preserved even after tumor resection (ibid.). Likewise, tasks involving “higher” cognitive functions activate areas in fMRI which are normally not activated (such as BA 46 for language) (ibid.). The brain’s dynamic is impressive as intracerebral functional reorganization may be observed even during a brain surgery (ibid.). The same has been shown in numerous experiments on animals (Hardcastle and Stewart, 2005; Anderson and Finlay, 2014), a source of evidence that had already influenced the way in which Goldstein interpreted his clinical observations.^1^

## The idea of degeneracy

As Goldstein already observed, a brain damage not only leads to “direct” (local and global) but also to “indirect” modifications, which reflect the reactions of the whole organism to the changes. Eventually, these two types of changes create a new mélange which can hardly be disassembled (see also Ben Yishay, 1996; Prigatano, 2011). As already mentioned, this whole process is driven by the organism’s tendency to deal with these changes and to re-establish a new meaningful relationship with its *Umwelt*. Although Goldstein’s interpretation cannot be reduced to evolutionary arguments (for reasons that cannot be explicated here), it alludes to the more general idea that biological systems have an intrinsic ability to maintain functions in the course of structural changes, at least to a certain degree (Kitano, 2004). Specific functions can obviously be constituted on the basis of structurally different elements, a biological property that is referred to under the term *degeneracy* (Edelman and Gally, 2001). It can be observed on different levels of analysis of biological systems, such as genes, cells (neurons), organs (brains), (super-)organisms etc. Even unicellular organisms easily change their molecular structure inherently and autonomously for the sake of function (von Uexküll, 1909; Fitch, 2008). This gives them the flexibility to adapt to changing environmental circumstances and to maintain or reproduce their identity (Varela, 1997; Thompson, 2007). In that respect, they differ fundamentally from technical artifacts.

The problem that brain areas activated in neuroimaging might not be necessary for a task, is rarely—but increasingly—accounted for in CNS by directly referring to the degeneracy concept (Price and Friston, 2002; Noppeney et al., 2004; Friston and Price, 2011). It is acknowledged that one and the same response may be produced by structurally different neural systems, and that this may account for the frequent finding that areas which are activated in fMRI during a task do not necessarily affect performance in the same task when lesioned. Noppeney et al. (2004) distinguish degeneracy *between* from degeneracy *within* subjects: degeneracy *between* subjects would explain why the same lesion/activation leads to different outcomes or why the same outcome can be caused by different lesions/activations in different subjects. At least on an ontogenetic scale, this kind of degeneracy seems to be enormous. This is shown in a range of clinical examples of normal cognitive-affective development despite severe lesions in childhood, such as tumor and hydrocephalus, even to the point of very little brain mass (Lewin, 1980; Borgstein and Grootendorst, 2002; Feuillet et al., 2007). High degrees of degeneracy become also manifest in the neurohistology (Amunts et al., 2000) and functional neuroanatomy (Hamilton et al., 2000; Burton et al., 2002) of even very “basic” motor and sensory functions, such as vision. Although there are developmental brain anomalies that cannot be coped with, developmental plasticity is so enormous that it seems rather unclear how it could inform us about localization, at least unless the development is known in closest detail. This is also reflected in the frequent finding that similar lesions or diseases of the brain can lead to vastly diverging ranges of impairment, depending on the amount of *cognitive reserve* or *brain reserve* (Stern, 2002). Interindividual degeneracy seems a highly relevant, though often neglected factor at least in earlier imaging studies, as differences in activation between subjects are oftentimes treated as unsystematic (error) variance, although this variance probably contains very important information (Price and Friston, 2002; Zilles and Amunts, 2013).

## Modularity: a must-have?

Degeneracy *within* subjects seems even more relevant for our discussion. As already mentioned in reference to examples of slow-growing tumors (Desmurget et al., 2007), it can be frequently observed in brain diseases, to rather impressive extents. It is, however, not restricted to clinical cases, but also seen in experimental imaging studies on repeated intra-day measurements, in resting conditions (Blautzik et al., 2013) as well as in experimental tasks (Gorfine and Zisapel, 2009). Methodically, it relates to the question of reliability of fMRI measurements (McGonigle, 2012) as well as to the interpretation of activations: a *causal* role of a brain region to an experimental effect cannot be demonstrated on the basis of *correlative* (fMRI) evidence alone. This is because lesions in an area activated in fMRI in response to a task might not affect performance in the same task, as one or more other areas might “do the job” equally well. As a solution, it has been proposed that lesion and imaging studies should be combined in determining both necessary and sufficient areas for a task (Price and Friston, 2002). Accordingly, areas should be classified with respect to their degree of degeneracy for a certain task, that is, the “minimum number of areas that must be removed before a function is lost” (ibid., p. 416). Although this proposal is a progress in comparison to earlier attempts of uncritical mapping, it still faces problems: it seems still largely based on a static view of the brain according to which lesions affect functions specifically. In other words, it still presupposes “modularity and segregation of sensorimotor and cognitive functions” (Price and Friston, 2002, p. 417). Here, even networks remain more or less static arrangements comparable to clockworks, collections of fixed and rigid gadgets, as a result of how the phenomenon is investigated. More generally, this becomes obvious regarding the notion of a “brain mechanism”, a common, widely used notion in CNS.

Goldstein (1934, Chapter 2) already criticized Charles Sherrington (1857–1952) for claiming that reflexes were perfectly rigid, uniform and repeatable, literally like the mechanical working of a steam engine. Goldstein nicely argued that Sherrington’s interpretation was bound to an isolation of the reflex arc, both experimentally and conceptually, from the organismic context. Within the context of these factors, the reflex response may greatly vary, depending on the “meaning” of stimuli, hormonal influences, state of arousal of the organism, postural changes etc. Goldstein did not mean to invalidate reflexes as important clinical signs, but to demonstrate that reflexes do not make good mechanisms, at least in the 19th century definition of the term. It is not my aim to discuss the different (and often vague) meanings of the term “mechanism” in CNS. In fact, this term has also been used in a pragmatic sense, i.e., as describing a causal relation between entities, a relation which may provide a starting point for further productive research efforts both on “higher” and “lower” levels of analysis (cf. Craver, 2005, 2006). Instead, I want to pick up Goldstein’s interpretation that the uniformity of phenomena may also result from the method of investigation. Could it not be that the way in which experiments are constructed in CNS also creates the impression of brain processes being more stable and fixed than they actually are? It is in fact a common advice for lab greenhorns that when conducting (neuro-)psychological experiments: (a) never to run too many trials of the same condition in one and the same subject; and (b) never to let one and the same subject take part more than once in experiments with very similar manipulations, as effects are soon reduced or even washed out (Bates and Dick, 2000). Why? The brain is deprived of the possibility to do what it permanently does in everyday life and for which it was designed: to learn and to adapt to permanently changing conditions. Interestingly, the idea that brain processes are highly stable shows up not only in proponents of imaging research, but also on the side of their critics, for example in the debate on the reliability of fMRI data (McGonigle, 2012). The widespread assumption of fixed and repeatable processes tends to be trustworthy; however, one does need to consider its limitations more seriously. These limitations become most obvious in a dynamical systems view on us as *Umwelt*-embedded living beings (see also Thompson, 2007, 341ff.).

## Is equipotentialism the answer?

There is not enough space here to discuss the idea of a specific localization of cognitive functions and I would not dare to claim that I can disconfirm it. In fact, it might be a useful heuristic to design experimental studies, but it seems so dependent on top-down assumptions and prone to immunization, that it can hardly be seen as a fact, and indeed resembles a “holy grail” (Friston and Price, 2011, p. 249).^2^ Things would be easier if we were able to independently identify invariant, context-independent basic entities on both sides (neural and mental) which could then be related to each other empirically. However, it seems rather unclear how this could be achieved. It seems improbable for the additional reason that the respective scientific concepts have very different historical and methodological origins. Accepting that these two levels of description might to some extent be incompatible may not be so bad after all as it leaves us with more scientific as well as clinical approaches than a localizationist success. At least, the ubiquitous conclusion in many CNS studies of the sort “area A is responsible for function F” seems underspecified: “Responsible” neither seems to mean necessary nor sufficient, at least in a considerable number of cases. As clinical evidence reveals, a view of dynamic, distributed brain activity seems much more adequate, where patterns of activation are more important than activations of particular regions (Bates and Dick, 2000; McIntosh, 2000; Knight, 2007) and where one and the same neuron might fire in different ensembles of other neurons, depending on the context (*transient plasticity*, McIntosh, 2000; or *reuse*, Anderson, 2010).

Is there no specialization in the brain and is thus equipotentialism (Lashley, 1950) the answer? In fact, Goldstein cannot be allocated unambiguously to one side of the localization debate. Considering him as an equipotentialist would wrongly assume that he did not see the clinical usefulness of lesion-deficit correlations. More importantly, it would mean to stick to a brain-immanent conception of function. His own approach seems to be much better described as the outcome of a dialectic process, as a synthesis between the two sides on a superordinate level (Wolfe, 2010): the changes due to a lesion can only be adequately captured on a higher level of description, namely one in which the relationship between the defect and the whole organism is decisive. In other words, a function is something that cannot be investigated in an appropriate way by just looking at the level of the brain.

In fact, Goldstein (1927, 645ff.) proposed a rather interesting and—at least for his time—revolutionary view about the brain, which he conceived of as a “Netzwerk” (network) with explicitly systemic properties: the brain is *always* active and incoming stimuli shift the distribution of activation within this network (rather than create it). The brain then attempts to return to its usual activation states as some sort of individual constants. Goldstein postulated such constants for all kinds of physiological processes in the body, as a means to maintain the organism’s identity. Some stimuli do not induce changes in activation, they are irrelevant. Those that do are incorporated into a larger system *including* the brain (and body). Lesions do not just change local activation, but they shift activation patterns globally, thereby leading to overall *qualitative* modifications within the system. These modified patterns can then be influenced in new ways by incoming stimuli, also inducing new phenomena. Referring to Gestalt ideas, lesions change the distinctiveness of foreground to background activations, thereby rendering processing of stimuli more unstable, undifferentiated, primitive and inflexible (Goldstein, 1927).

Apparently, Goldstein ingeniously anticipated some very modern assumptions of dynamical systems theory including the idea of *attractors* or *steady states* (Friston, 1997; Sporns, 2009). Here, mental functions are seen to depend upon complex, distributed networks (Sporns, 2009). Brains can maintain different states simultaneously, and may switch between them depending on external or internal perturbation (“multistability”, Tognoli and Kelso, 2014). Lesions lead to overall system changes and to the creation of new stable states (Sporns, 2009). In dynamical systems theory, global, qualitative changes can be accounted for straightforwardly, as they result from bifurcations induced by changes in control parameters. Such a view is nevertheless compatible with the idea that neurons and networks are linked to some mental abilities more closely than to others, due to differences in connectivity with other central or peripheral networks. Such systems could even entail *Umwelt* properties such as the subjective experience of stimuli as control parameters of the system’s dynamics (Thompson and Varela, 2001), very much in the sense of Goldstein (1927, p. 646). Without going into detail, a dynamical systems view of the brain seems to be much more appropriate, especially with respect to the pervasive clinical observation of global changes which do not seem to be sufficiently accounted for on the basis of a modular lesion-deficit approach.

## Is sick just different?

As discussed above, there are interesting attempts to take the concept of degeneracy into account, by combining data from brain-lesioned patients and imaging data on healthy subjects (Price and Friston, 2002; Noppeney et al., 2004). However, the concept of a “deficit” (Price and Friston, 2002, p. 419) seems all too clear here, as does the concept of “clinical data” (Kandel et al., 2013, p. 661) or “brain diseases” (Kandel et al., 2013, p. 659). Is this indeed the case?

Any attempt to define a deficit leads to the question of how strong a particular data point should deviate statistically from an average in order to classify it as abnormal. In the end, this issue is a matter of convention. However, no such agreement answers the clinical question: what makes a deviance *pathological*? Is performance as such the crucial place to look in order to find an answer to this question? Goldstein has shown that this question is indeed intricate: first of all, it seems to be intuitively clear that deviance refers to a purely quantitative analysis. Even though deviance seems to be associated with disease, the former is not sufficient for the latter. If it were sufficient, all deviance would imply a disease, but as the medical notion of “normal variant” shows, this is clearly not the case. There are numerous e.g., anatomical peculiarities which clearly deviate from an average, but are not seen as pathological (e.g., missing wisdom teeth). Even though some of these cases are controversial, the fact that there can be a controversy at all demonstrates that a deviance in itself does not seem to give a clear definition. Deviance does not even seem to be *necessary* to speak of a deficit: there are e.g., many brain-damaged patients who deviate clearly from an average in specific tasks, but get along well in their everyday life. Thus, what makes a deviance pathological and calls for treatment? Goldstein’s answer: when there is an imbalance between the organism and its *Umwelt*, between possibilities and demands, an imbalance that cannot be overcome, this is where suffering and treatment begin. Thus, “deficit” is not just *something inside* people (“produced” inside their brains), but can only be understood from the perspective of a higher order *relationship*. The aim of any treatment is to establish a new state of balance which relies, to a considerable extent, on the subjective perspective of the patient. The primary aim of any treatment is to enable a maximum of vital interaction between organism and *Umwelt*, a maximum of *responsivity* (Goldstein, 1934), in which an organism can unfold its potential. Hence, diagnosis and treatment of brain-related deficits includes reference to: (i) a relational (organism-*Umwelt*) perspective; and (ii) to *individual* norms, in analogy to many other bodily processes (Goldstein, 1934). Issues (i) and (ii) are taken into account by most therapists as a matter of course, but seem to escape many researchers when working on “clinical data”. However, all these issues seem not only relevant for therapy, but for all scientific studies that make reference to brain damage.

## Personal, situated encounters: a comprehensive view of clinical neuropsychology

Even from the perspective of modern clinical-neuropsychological practice, Goldstein’s ideas still bear great significance. This is especially true for his idea that quantified results crucially need qualifying information from the personal and situational context in order to be adequately interpreted. In fact, neuropsychological assessment is a much more complex and integrative enterprise than is conveyed in the lesion-deficit view of CNS as depicted above. Essentially, neuropsychological test scores result from an interaction between patient and examiner (Lezak et al., 2004). Thus, it would be oversimplified to interpret scores as absolute and in isolation, i.e., by blinding out the context of their origin (ibid., p. 108). Test scores are over- and underspecified at the same time (Lezak et al., 2004): on the one hand, tests are designed for rather specific aspects, they can hardly be used to capture global changes, such as in overall behavior or personality. On the other hand, test scores are very complex entities, influenced by a diversity of factors. Therefore, they have to be *validated* (cf. Goldstein and Scheerer, 1941).

Validating information can come from a variety of sources (cf. Lezak et al., 2004): the medical history of the patient, the patient’s premorbid status (occupational demands, abilities, comorbidities etc.), his/her subjective experience of the changes, observations from the side of the patient’s family etc. During the assessment proper, further important sources of information include: the patient’s general attitude towards the examination and towards his/her problems (insight into/reaction to deficits, test anxiety, motivational status), the influence of overlaying global changes (apathy, concretism, inflexibility, disinhibition), qualitative aspects of test behavior that are hard to embrace in a standardized way (bizarre performance, self-comments, enriching solutions, profit from help etc.) and observations of behavior in real-world situations (see following sections). In this process, data from the third-person perspective (such as the test scores or behavioral observations) are complemented by the patient’s subjective experience (first-person perspective) as well as the experience of the examiner in the empathic interaction with the patient (second-person perspective). Both first- and second-person perspectives become even more relevant in neuropsychological therapy: including them has proved to embrace the multiple changes induced by a brain lesion much better (Ben Yishay, 2008; Gracey et al., 2008; Yeates et al., 2008) and to contribute essentially to therapeutic success (Prigatano et al., 1994; Klonoff et al., 2001; Wilson, 2010, 2011). All these studies converge in the view that therapeutic success is most appropriately defined as (re-)establishing an identity and a world of meaning in which patients can realize their possibilities as best as possible (Wilson, 2010; Prigatano, 2013).

I do not want to argue that test scores should never be used to address more general hypotheses (Frisch et al., 2013), but that we should always keep in mind that doing so is a highly abstract enterprise which intentionally blinds out a lot of qualifying information. Imagine the loss of information that results from teachers’ attempts to summarize all of a pupil’s performance, abilities, possibilities, personal problems etc. in the course of a school year in a few school grades. This does not mean that grades say nothing at all. Quantifying is also important in clinical contexts, but if clinicians have nothing else than numbers, they will miss very crucial things about the brain (and will hardly be able to help the patient). Thus, sticking to a lesion-deficit model provides a much more limited picture of the nature of brain damage in contrast to clinical-neuropsychological practice. A pure lesion-deficit perspective may suggest that brain damage just impairs functions, but actually, it changes a *world* (Luria, 1987). Therefore, clinical practice crucially relies on information from all three perspectives, 1st, 2nd and 3rd person. None of them can be claimed superior *a priori* and none of them should be taken at face value. Information from all three sources is critically integrated on the basis of theoretical knowledge, practical experience and intuition. Thus, clinical-neuropsychological practice transcends the lesion-deficit model considerably. Therefore, it seems hasty to applaud clinical neuropsychologists for allegedly relying on a third-person lesion-deficit approach as a “serious, scientific way” of study (Dennett, 2003, p. 22), as this does not well capture clinical work. The first-person perspective is essential in understanding a brain disorder. Any disorder is first and foremost something that is *experienced* (Toombs, 1995, 2001; Varela, 2001). First-person information is the clinical practitioners’ “daily bread and butter” (Cytowic, 2003, p. 165) and sometimes the only reliable information that we can get on a patient. This may hold for the second-person perspective as well, especially with respect to the importance of empathy (Toombs, 2001). To understand a brain lesion (and to treat the person who has it), both perspectives are indispensable. There are many clinical phenomena that could not be scientifically studied without them, such as pain (cf. Velmans, 2007), but also perception (see below; cf. Feest, in press). In addition, they are the entry gate for therapeutic interventions (Varela and Shear, 1999; Prigatano, 2003, 2011). This does not mean that they are always taken at face value, as they are validated using information from other sources (second- and third-person) (Varela and Shear, 1999; Jack and Roepstorff, 2002; Velmans, 2007), but this is true for these other sources vice versa (Jack and Roepstorff, 2002, 2003). One could also say that a situated encounter between subjects is the ultimate interpretational frame for clinical-neuropsychological practice.

Despite the limitations of the lesion-deficit model, it seems to be desired by neighboring disciplines such as psychiatry (Kandel, 1998; Insel and Quirion, 2005; Kandel et al., 2013). Neurology is admired for its alleged success in describing diseases as “pure mechanical, brute (…) affections of the brain” (Graham, 2013, after Walter, 2013, p. 7). However, from the view that I have developed here, neurological disorders seem to be much less “brain-inherent” than the above characterizations might suggest. At first glance, it seems self-evident that mental changes such as in Alzheimer’s disease (AD) are directly related to pathological changes in the brain. Despite strong correlations between pathological burden and clinical deficits, however, these correlations are far from complete, and there are even individuals who do not show any measurable cognitive decline despite extensive AD related pathology (Riley et al., 2002). Such discrepancies can be explained by differences in the compensatory abilities of individuals, or rather individual brains (Stern, 2002), but this interpretation points to two conclusions: first, it presupposes a systemic view of the brain, as compensation entails that the brain’s capacities have to be reorganized in some way or another. Second, it presupposes a tight coupling with the environmental context in which a discrepancy is somehow *experienced*. These are crucial reference points for any compensational efforts. Thus, an integrative, embodied perspective onto these issues (cf. Walter, 2013) seems much more helpful also for psychiatry than narrowing them down according to a lesion-deficit ideal.

## The experimental paradigm in human neuroscience: the one and only?

One might question the general usefulness of clinical-neuropsychological data for a scientific study of mind and brain, arguing that they are seemingly too complex. Why not stick to the good old experimental method when investigating brain lesions, which seems to provide us with much more specific and objective results? Certainly, I do not want to argue against the general utility of experimental methods in studying mental phenomena. Especially in the area of philosophy of mind, however, there seems to be a strong preference for the assumption that the experimental method, which has proved so useful in other areas of science, can be transferred directly to the study of the mind (Dennett, 2003). Experiments may even be treated as a gold standard to investigate the mind scientifically, as “it isn’t science until you can turn it into …. experiments” (Dennett, 2003, p. 23). They are often understood to be powerful enough to answer even metaphysical questions, such as questions of the self, morality, freedom of will etc. (see Churchland, 2005, 2008; Metzinger, 2009; Kandel et al., 2013). This goes hand in hand with a strong emphasis on a third-person perspective as being scientific and “real”, while outranging first- and second-person perspectives, at least as long as the latter cannot be translated into a third-person view (Dennett, 2003; Churchland, 2005; see also Jack and Roepstorff, 2002, 2003). Thus, it is not so much the method itself, but first and foremost the way in which it seems to be decontextualized and overemphasized that deserves critical discussion.

Regarding the natural sciences in general, philosophy of science has convincingly uttered doubts about the radically objective nature of experiments and concluded that experiments cannot establish a complete independence between the observer and the object under study. A major source for this conclusion comes from 20th century physics (Primas, 1994). In short, there is something we put into an experiment and something we get out, and it is not possible to completely separate one from the other (Harré, 2003). Taking this for granted, however, one might nevertheless still postulate that the experimental method should work in studying mind and brain straightforwardly (Dennett, 2003). But does it?

As we have seen, Goldstein doubted that the experimental method from physics and chemistry worked equally well in the area of biology. He insisted that the isolation of biological phenomena as a result of an experimental manipulation runs the risk of giving a distorted picture of the living. For example, in order to study animals experimentally, we have to interfere massively with their genuine state of existence by taking them out of their natural environment (*Umwelt*, as part of the organism), to the point of even killing them to reveal their anatomical and physiological details. In other words, we deprive them of the very property that we intend to study: life. Especially in the area of life, we do not just *find* phenomena, we *create* them to a considerable extent when adopting an experimental procedure. This problem had also been emphasized in modern physics (Bohr, 1933). In addition to research on plants or animals, there is a further interesting property of humans, namely, that they are interpreting, interacting subjects. This aspect is blinded out more or less intentionally in lab experiments, as we only want to have “objective” results in science that are not contaminated by anything purely “subjective” (Dennett, 2003). However, this idea from the classical natural sciences becomes very intricate when applied to research on humans: in all kinds of psychological experiments, it turns out to be important to ask the subjects afterwards about additional variables, such as the strategies applied, whether the experiment was short and interesting enough to stay concentrated, which hypotheses they came up with about the study, etc. (Jack and Roepstorff, 2002). In addition to this first-person perspective, experimenters often rely on their own intuition about their subjects’ attitudes and strategies, on the basis of their interaction with the subject (that is, from a second-person perspective). All this information is crucial to design experimental paradigms and to interpret results (Jack and Roepstorff, 2002).

The fact that we often have to rely upon information from the first- and second-person-perspective to do psychological experiments, but generally disregard it as being not scientific reveals the highly ambivalent nature of the experimental method in research on humans. This aspect is already entailed in Goldstein’s *projective* notion of organism (Wolfe, 2010): humans cannot encounter each other other than as “organisms” (subjects), as participants in an interaction. This holds true not only for the clinician, but also for the experimenter (not to mention all sociological issues in actual scientific practice). By seeing others as subjects, the researcher has to see himself as a subject as well. Otherwise, no interaction, and thus no psychological research, would ever be possible. From a Goldsteinian view, one might say that the third-person perspective presupposes a second-person perspective and that the former is derived from the latter in a way (see also Thompson, 2007, 309ff.). The “objectivity” of psychological experiments relies on an artificial exclusion of these factors. Experimental arrangements are even *designed* to blind out these influences. This can indeed be a useful scientific strategy. However, it creates great confusion if it is not remembered as being a strategy, and if it is claimed to produce results that are independent from the notion of a subject. Human subjects *know* that they are in an experiment and what experiments are about, they anticipate goals and hypotheses, they interact with the experimenter on different levels, they want to help science or resist to being manipulated etc. These issues have been thoroughly and extensively investigated in experimental social psychology for decades (Strohmetz, 2008). Interestingly, the influence of these factors is almost never systematically addressed or even discussed in CNS, although it might also considerably contribute to the problem of diverging and non-replicable results across studies.

## Cognitive neuroscience in a vat

To isolate phenomena experimentally is a way to study them, but it creates problems, as we have seen. A serious problem, which follows from a contextual isolation, and which arises from Goldstein’s emphasis on the entanglement between organism and *Umwelt*, is *external* (or *ecological*) *validity*, a concept which is most often neglected in favor of internal validity in CNS. Usually, there is much more space in the discussion sections of journal articles devoted to discussions of whether an experimental effect was in fact caused by the factors as argued, whereas it is mostly just presupposed that the results are equally valid outside the experimental setting. For example, it is often taken for granted that a short-term memory task in the scanner (e.g., an n-back task) can be unequivocally transferred to short-term memory performance in real life (e.g., putting different appointments of a day into a reasonable order). This might well be the case, but the problem is that it is rarely shown or even put up for discussion. Studies comparing performances in a specific experimental task inside and outside the *scanner* (Koch et al., 2003; Koten et al., 2013) seem to converge on the view that the general pattern of behavioral results is comparable, but that there are overall differences (e.g., in response latencies). Obviously, the scanner seems to create a special experimental environment compared to the usual lab setting, and it remains unclear how this affects imaging results. However, this discussion misses an important point which outranges the actual problem considerably: it is not the comparison between different kinds of *experimental settings*, but between experimental settings and *real world situations*. Thus, it is less the scanner than the experimental isolation from the environmental context that seems to create a problem.

Clinical neuropsychology, by contrast, has to have an emphasis on ecological validity (Shallice and Burgess, 1991; Manchester et al., 2004; Frisch et al., 2012), for different reasons. First of all, any therapeutic attempt aims at helping a patient to cope with his everyday life challenges, not at solving problems in abstract cognitive tasks. Secondly, the real world is an important way to validate neuropsychological test results, to judge whether they actually capture the problems of a patient (as revealed from other sources of information). Although the construction of neuropsychological tests is much more geared to the real world than experimental tasks, they are never taken as exact simulations of real world conditions. Take, for example, executive functions, the ability to plan, initiate and monitor actions. As Shallice and Burgess (1991) have shown in a seminal paper, “classical” executive tests (such as Stroop, Planning, Categorization, Verbal Fluency, Switching etc.) may reveal normal results in brain-damaged patients with massive executive problems in everyday life. This led to further research on the kind of problems that these patient suffered from (“multitasking”) and to the development of new tasks to capture them (Shallice and Burgess, 1991; Burgess, 2000), thereby constantly accounting for the external validity of these measures (Alderman et al., 2003; Cuberos-Urbano et al., 2013). This kind of research nicely supports Goldstein’s conclusion that the real world, i.e., the relationship of individuals to the demands of their specific *Umwelt*, is the crucial reference point from which the brain should be studied. However, experimental neuroscience does not seem to have such a corrective. As long as experimental evidence is only validated through other experimental evidence, this kind of research can hardly dispose of the criticism that it produces results that are detached from the “real world” and based on a principle of constant self-reference. Instead, it seems necessary to enhance the ecological validity of studies, e.g., by more forcefully including behavioral observations or virtual reality paradigms, to name just two examples.

## The work bench engine view of the brain

One might object that the Shallice and Burgess (1991) example only demonstrates that the everyday life context is important to find appropriate tasks. One could still argue that existing experimental results can be generalized to real world situations unequivocally. Is there any evidence against this assumption? Goldstein proposed, with reference to Fuchs’ (1992) findings on hemianopic patients described above, that even if we can exactly determine the blind areas in the visual field, this “does not tell us anything about the function of the calcarine fissure in real life vision” (Goldstein, 1926, p. 87, my translation). The determination of visual field defects is the result of an isolating procedure (perimetry). This is important for diagnostic reasons, but it is “construed” in some way. This becomes obvious if we turn to a setting which is a little more natural, such as the studies of Fuchs (1922). Here, the visual field defect becomes more variable, as the fovea may dynamically shift into different parts of the periphery in order to preserve the focus of vision in the center of the visual field. In the case of such a “pseudofovea” (Crossland et al., 2007; Kuhn et al., 2012), anatomy seems to step back in favor of function, that is, coming along in the respective *Umwelt* as best as possible. Anatomically, this may be reflected by top-down influences of “higher” cortical areas to the visual cortex (Varela et al., 1991). Something like a pseudofovea only develops *for* and *in* an active interaction with the environment (Noë, 2009).

In fact, it is very surprising how much literature e.g., on vision is devoted to meticulous descriptions of the respective anatomy (e.g., retinotopic/tonotopic maps, see Thompson and Toga, 2003), which guides the explanation of vision defects after brain damage (Zhang et al., 2006). There exist, however, almost no systematic reports on how the visual *experience* changes in the course of a brain lesion, and how this affects actions of the organism in its *Umwelt*. Even in the case of extended visual field defects (such as hemianopia), these defects are normally not experienced in the way that they come out in a perimetric examination. As an example, patients with hemianopia rarely report that half of their visual field is missing nor do they act that way, although they normally do notice many problems such as overlooking objects etc. Even patients who are trained neurologists have to apply a specific testing procedure, e.g., by slowly moving fingers from the periphery to the center of the visual field, in order to determine the actual changes (Cole, 1999)! Each of us lives with a “natural” visual field defect, the blind spot, which we normally do not experience and which we can only reveal by also applying a specific procedure, such as moving two fingers in a certain distance from each other in front of our eyes. Would we conclude, however, that the results of this procedure (namely, that one of the fingers disappears at some point) is how we *normally* see? That they demonstrate us our “actual”, “real” way of seeing, even if we normally do not know this? Likewise, would we conclude that the perimetry reveals how the hemianopic patients *really* see? “Normal”or “real”, relative to what?

In sum, these examples again show that the first-person perspective (here, visual experience) provides substantial evidence to find out interesting issues about brain lesions, and brains (Varela and Shear, 1999; Cytowic, 2003; Thompson, 2007). This does not mean that first-person data *necessarily* increase ecological validity of experimental settings (cf. Feest, in press), but this point seems to be beyond the scope of the present paper. Furthermore, examples such as the one of the pseudofovea nicely show how tightly perception and action are coupled within the organism-*Umwelt* context (Thompson, 2007; Noë, 2009). Isolating phenomena in a lab setting (here including perimetry) may reveal interesting results, but it seems problematic to conclude that these results are necessarily “more real”. As an analogy, even if a car engine is tested and described as thoroughly and in as much detail as possible, we may not yet know very much about how the car drives.

## Conclusion

In mid-19th century Germany, a group of scientists aimed at establishing materialism in natural science, also with respect to the mind. They conceived mental phenomena as some sort of bodily secretion. As an analogy, the brain was seen as a producer of thoughts just as the kidney was seen as a producer of urine (Moleschott, 1852/1971; see also Hagner, 2008). Although brain-kidney (or mind-urine) analogies like these are certainly not maintained anymore in such a crude form, the general idea of the brain as a “mind-producer” still seems to be very much *en vogue*. Mind and consciousness are often seen as located *inside* the head, being a product of the brain’s computational power (Kandel et al., 2013). Higher-order concepts such as person/organism, meaning or interaction are then considered as secondary notions resulting from the interplay of basic neural processes which can be sufficiently identified from a third-person, experimental perspective as neuroscience’s *sine qua non*.

It is impossible to discuss all the complex and intricate philosophical and empirical issues that are connected with these claims and to judge them in their entirety in one paper. Nevertheless, I have tried to provide a number of arguments from clinical neuropsychology that question these assumptions, contrary to widespread intuitions in the field. I am convinced that CNS would profit from incorporating views of the brain that quickly become apparent when encountering brain-damaged individuals in clinical-neuropsychological assessment and therapy, such as: (i) Brain, organism and *Umwelt* are deeply intertwined and interrelated, they constitute a global, highly dynamic system; (ii) mind is constituted within this interwoven system, from which it cannot be reasonably isolated in scientific research; (iii) living systems cannot be fully decomposed into independent, basic units of explanation (such as brain modules, neurons, genes etc.), but can only be understood by assuming a non-transparent interrelation between supervening whole and lower-scale components; (iv) living systems are autonomous agents, actively maintaining an identity within an experienced, meaningful *Umwelt*, thereby adapting to perturbations and intrinsically generating norms; and (v) experimental methods in research on mind and brain are limited in their validity; they should therefore be complemented with information not only from more ecological third-person approaches (e.g., real-world behavior), but especially from first- and second-person perspectives in order to validate and enrich them.

All these ideas follow from Kurt Goldstein’s clinical and theoretical work, which proves still significant, despite its age and its—to modern eyes—antique terminology. Notably, ideas such as those in (i) to (v) are central to the embodiment approach in cognitive and neuroscience (Varela et al., 1991; Varela, 1997; Clark, 1999; Thompson and Varela, 2001; Thompson, 2007) which, at least from my clinical-neuropsychological perspective, seems to be much more appropriate for brain-mind research than concurring isolationist and computationalist approaches. I would like to explicitly emphasize and acknowledge Goldstein’s profound contributions to the embodiment approach which are obvious not only for theoretical, but also for historical reasons.^3^ This might enrich as well as strengthen the embodiment endeavor. Especially the fact that Goldstein developed most of his concepts in his clinical work with brain-damaged individuals seems particularly interesting in this respect. Conversely, I hope that these ideas, when spreading in CNS, could act back into the clinical disciplines, to enhance basic research’s practical benefit, which in the eyes of many practitioners is largely overestimated (Wilson, 2005). This could lead to more appropriate principles in the education of medical and psychological practitioners, which is often dominated by isolationist, third-person views. Last but not least, we should be aware that overemphasizing such views may have disastrous consequences for clinical practice (Toombs, 2001).

Is it the core problem to relate the more global clinical perspective to the more fine-grained view of experimental CNS? This cuts the story too short, as the latter seems to draw from several problematic assumptions. Thus, there is no one-way street: clinical practice does not just add to laboratory data, it can provide an interesting alternative perspective in its own right, which we would not find when sticking to experimental studies on healthy individuals. Clinical neuropsychology beyond the lesion-deficit-model could provide a more appropriate general research paradigm for CNS. However, progress can only be achieved when we dare to put even those assumptions into doubt that seem undoubted. Here, we should welcome external perspectives from interdisciplinary sources, such as the humanities, but also from modern physics. In fact, this seems to be the “strongest strength” of science and the only way to nourish creative power: to be prepared to give up even the core assumptions if they turn out to be questionable, and, most importantly, to dare to keep the questions open.

## Conflict of interest statement

The author declares that the research was conducted in the absence of any commercial or financial relationships that could be construed as a potential conflict of interest.

## Footnotes

^1^It seems noteworthy that Goldstein (1934) made extensive reference to experimental studies on animals, especially those by Albrecht Bethe (1872–1954), a disciple of Friedrich Goltz (1934–1902). Bethe worked as a physiologist at Frankfurt University at the same time as Goldstein. He demonstrated that the amputation of extremities in animals such as insects, crab and mammals led to a re-coordination of movements in a way that came as close as possible to the abilities before the damage. Bethe demonstrated that this adaptation occurred immediately and spontaneously (that is, without learning), independent of higher (cortical) control and in a way that could not have been pre-programmed (Bethe, 1933). Similar to Goldstein, Bethe originally came from a localizationist tradition, which he abandoned in the course of his experimental work. He advocated a systemic view of the brain and criticized to speak of a “function as if it were contained in a box” (Bethe, 1933, p. 218, my transl.).

^2^Friston and Price (2011, p. 242) put forward independent evidence from network theory (Sporns, 2009), which has emphasized the importance of modularity in networks as a means of robustness (Kitano, 2004), to protect against external perturbations (such as lesions). With this interpretation, however, Friston and Price seem to equate neural modularity with cognitive modularity from the beginning. By contrast, Sporns (2009, p. 195) explicitly distinguishes between the two notions of modularity. In fact, the idea of mental modules being identical to neural modules seems to undermine the very idea of robustness rather than to corroborate it. Furthermore, claims in favor of modularity from an evolutionary perspective (selective structural changes in response to functional adaptations) seem very appealing at first sight, though much less supported by empirical evidence as one might think (see Anderson and Finlay, 2014). On the contrary, despite interesting exceptions, data seem to support the view that neurons engage in multiple, diverse behaviors in very different kinds of species (ibid.).

^3^The embodiment approach makes extensive reference to the ideas of two philosophers, among others, namely Maurice Merleau-Ponty (Varela et al., 1991; Thompson, 2007) and Hans Jonas (Weber and Varela, 2002; Thompson, 2007). A central figure in this respect is the philosopher Aron Gurwitsch, a disciple of Edmund Husserl from 1922 onwards. Gurwitsch came to Frankfurt a few years later to work together with Kurt Goldstein and his most important collaborator, the Gestalt Psychologist Adhemar Gelb (esp. to work on Gelb and Goldsteins concept of a loss of “abstract attitude” in the aftermath of brain lesions). Gurwitsch had to flee Nazi Germany in 1933 to Paris. Here, he gave lectures at the Sorbonne University on Phenomenology and Gestalt Psychology, which Merleau-Ponty attended (Gurwitsch and Embree, 2010). Both met frequently and Merleau-Ponty was introduced to many of Goldstein’s ideas, as is most clearly reflected in the main topics of his first major work (Merleau-Ponty, 1942/1967): Constitution of an *Umwelt* through constant self-actualization of an organism, holism in perception, reflex as an argument against atomistic theories of behavior, norm-giving as essential property of life, role of symbols in the human mind etc. (cf. Moran, 2002, 411ff.). Many of these issues coined the embodiment approach (Thompson, 2007, p. 147). Hans Jonas got to know both Gurwitsch (Jonas, 1973) and Goldstein (Jonas, 1959) already in Germany, and all three became colleagues in their shared U. S. exile (*The New School of Social Research*, New York). Hans Jonas, a disciple of Husserl and Heidegger, saw Goldstein as a major influence (Jonas, 1959) and important issues from his work (Jonas, 1966) seem to reflect this influence (e.g., life vs. machines, organismic identity and existential concern). Thus, it turns out that many ideas which fed into the embodiment approach are rooted in Goldstein’s clinical-neuropsychological observations. Many thanks to Alexandre Métraux for information on these issues.
